# Supplementary material for: Complexome profiling of the Chlamydomonas psb28 mutant reveals TEF5 as an early PSII assembly factor
Source: Plant Cell. 2025 Mar 17;37(6):koaf055. doi: 10.1093/plcell/koaf055 (PMC12164590; doi:10.1093/plcell/koaf055)
Supplement: koaf055_Supplementary_Data [file koaf055_supplementary_data.zip › psb28_interactive.zip!/plots/intensity/qualityPlots/peptides/PSBP2_Cre16.g678851.t1.1.html]

### Cre16.g678851.t1.1

Trivial name: PSBP2  
SeqCoverage [median]: 8.10  
Mass: 30.29 kDa  
  
 

| Sequence | MissedCleavage | Mass [Da] | wtIntensity | muIntensity | Score |
| --- | --- | --- | --- | --- | --- |
| ILNTVTIFNSR | 0 | 1276.71 | 0.00 | 400.08 | 65.35 |
| LYEYEYELDSTR | 0 | 1579.70 | 0.00 | 641.28 | 78.65 |
|  |  |  | 0.0 | 1041.4 |  |
